# Adjunctive Oral Olanzapine for the Prevention of Postoperative Nausea and Vomiting in Adults: A Systematic Review and Meta-Analysis of Randomized Controlled Trials

**DOI:** 10.7759/cureus.119626

**Published:** 2026-11-13

**Authors:** Lucas B Garcia, Ana J C Ferreira

**Affiliations:** 1 Internal Medicine, Universidade Federal do Paraná, Curitiba, BRA

**Keywords:** antiemetics, general anesthesia, meta-analysis, olanzapine, postoperative nausea and vomiting, systematic review

## Abstract

Olanzapine blocks several receptors involved in postoperative nausea and vomiting (PONV), but its incremental effect when added to otherwise identical antiemetic prophylaxis is uncertain. We systematically searched PubMed, Embase, and the Cochrane Central Register of Controlled Trials (CENTRAL) from inception to 16 October 2026 for randomized trials in adults undergoing general anesthesia that compared prophylactic oral olanzapine plus standard prophylaxis with the same prophylaxis without olanzapine. One reviewer screened records, extracted data, and assessed risk of bias (RoB 2) and certainty of evidence (Grading of Recommendations Assessment, Development and Evaluation (GRADE)). Risk ratios (RRs) were pooled with restricted maximum likelihood (REML) random-effects models and modified Hartung-Knapp confidence intervals (CIs). Of 108 unique records, six trials were included. Four trials (687 participants) contributed to a study-defined PONV-related composite within 24 hours: RR 0.59 (95% CI 0.34-1.01; I² = 60%; very low certainty). In one of these trials, the postoperative outcome used was declared post hoc by its authors, and in another the composite also counted rescue antiemetic use; excluding the latter in a post hoc sensitivity analysis gave RR 0.50 (0.26-0.98). In three trials (469 participants), olanzapine reduced nausea (RR 0.33, 0.12-0.91; low certainty) and rescue antiemetic use (RR 0.24, 0.06-0.98; low certainty), while the effect on vomiting was very uncertain (RR 0.25, 0.03-2.20; very low certainty). All trials had some concerns of bias. Adjunctive olanzapine may reduce postoperative nausea and rescue antiemetic use, but the evidence for the overall composite is very uncertain and does not exclude no effect. Larger trials with prespecified, uniform outcome definitions and systematic sedation reporting are needed.

## Introduction and background

Postoperative nausea and vomiting (PONV) remains one of the most frequent complications after general anesthesia; it delays recovery, increases the need for rescue antiemetics, and is a highly distressing experience associated with significant patient dissatisfaction [1]. Current consensus guidelines recommend multimodal prophylaxis for patients at increased risk [1]. Olanzapine, an atypical antipsychotic, antagonizes dopamine, serotonin, histamine, and muscarinic receptors and is established for chemotherapy-induced nausea and vomiting, which makes it a plausible adjunct for PONV [2]. Its main adverse effect in this setting, sedation, must be weighed against any antiemetic benefit and is discussed below.

Several randomized trials have tested preoperative oral olanzapine [3-8], and two meta-analyses have been published [9,10]. However, these trials asked different clinical questions: some added olanzapine to an unchanged background regimen, whereas others replaced standard prophylaxis with olanzapine [11]. Outcome windows also differed, with some trials measuring nausea and vomiting after hospital discharge rather than during the first postoperative day. The incremental value of olanzapine is best estimated from trials in which the only difference between groups is the addition of olanzapine.

We therefore conducted a systematic review and meta-analysis restricted to randomized trials that compared oral olanzapine added to standard antiemetic prophylaxis with the same prophylaxis without olanzapine, keeping postoperative and postdischarge outcomes separate.

## Review

Methods

This review is reported according to the Preferred Reporting Items for Systematic Reviews and Meta-Analyses (PRISMA) 2020 statement [12]; the completed checklist is provided in Appendix 3. The review was not prospectively registered, and no protocol was published. Eligibility criteria and the analysis plan were finalized after the authors had become aware of some of the included trials; deviations and post hoc decisions are reported explicitly.

Eligibility Criteria

We included randomized controlled trials enrolling adults undergoing surgery under general anesthesia in which a single preoperative oral dose of olanzapine was added to prophylactic antiemetic therapy that was otherwise identical in both groups (including placebo-controlled trials). Trials in which olanzapine replaced standard prophylaxis, used a different active comparator, lacked a concurrent control group, or primarily investigated another indication (for example, anxiolysis) were excluded. Trials reporting only postdischarge or recovery-room outcomes were eligible for narrative synthesis.

Information Sources and Search

PubMed (via the NCBI interface), Embase (via Embase.com), and Cochrane Central Register of Controlled Trials (CENTRAL) (via the Cochrane Library) were searched from the inception of each database to 16 October 2026 without restrictions on language, publication date, or study design. Strategies combined controlled vocabulary (MeSH in PubMed, Emtree in Embase) and free-text terms for olanzapine and for postoperative nausea or vomiting; the CENTRAL search used free-text terms in title, abstract, and keyword fields only. The full Boolean search strings for each database, as executed, are reported in Table 1. Trial registry records indexed in CENTRAL were screened, and the reference lists of previous systematic reviews [9,10] were checked.

**Table 1:** Search strategy for each database All searches were run without date or language restrictions. Other sources: reference lists of previous systematic reviews [9,10] and trial registry records indexed in CENTRAL. UTC−3: Coordinated Universal Time minus 3 hours (Brasília time); CENTRAL: Cochrane Central Register of Controlled Trials; MeSH: Medical Subject Headings; tiab: title/abstract field; /exp: Emtree term with explosion; ti,ab,kw: title, abstract and keyword fields; NEAR/5: terms within five words of each other; PONV: postoperative nausea and vomiting; PDNV: postdischarge nausea and vomiting

Database (interface)	Date and time of search (UTC−3)	Coverage	Limits	Full search string (as executed)	Records
PubMed (pubmed.ncbi.nlm.nih.gov)	16 October 2026, 21:17	Inception to 16 October 2026	None	("Olanzapine"[Mesh] OR olanzapine[tiab] OR zyprexa[tiab]) AND ("Postoperative Nausea and Vomiting"[Mesh] OR "postoperative nausea"[tiab] OR "post-operative nausea"[tiab] OR "postoperative vomiting"[tiab] OR "post-operative vomiting"[tiab] OR PONV[tiab] OR PDNV[tiab] OR ((postoperat*[tiab] OR post-operat*[tiab] OR postsurg*[tiab] OR post-surg*[tiab] OR perioperat*[tiab] OR peri-operat*[tiab] OR anesthe*[tiab] OR anaesthe*[tiab]) AND (nausea[tiab] OR vomit*[tiab] OR emesis[tiab] OR antiemetic*[tiab] OR anti-emetic*[tiab])))	25
Embase (Embase.com, Elsevier)	16 October 2026, 21:22	Inception to 16 October 2026	None	('olanzapine'/exp OR olanzapine:ti,ab,kw OR zyprexa:ti,ab,kw) AND ('postoperative nausea and vomiting'/exp OR 'postoperative nausea':ti,ab,kw OR 'post-operative nausea':ti,ab,kw OR 'postoperative vomiting':ti,ab,kw OR 'post-operative vomiting':ti,ab,kw OR ponv:ti,ab,kw OR pdnv:ti,ab,kw OR ((postoperat*:ti,ab,kw OR 'post-operat*':ti,ab,kw OR postsurg*:ti,ab,kw OR 'post-surg*':ti,ab,kw OR perioperat*:ti,ab,kw OR 'peri-operat*':ti,ab,kw OR anesthe*:ti,ab,kw OR anaesthe*:ti,ab,kw) AND (nausea:ti,ab,kw OR vomit*:ti,ab,kw OR emesis:ti,ab,kw OR antiemetic*:ti,ab,kw)))	81
CENTRAL (Cochrane Library, Advanced Search)	16 October 2026, 21:22–21:27	Inception to 16 October 2026	Trials	#1 (olanzapine OR zyprexa):ti,ab,kw #2 ("postoperative nausea" OR "post-operative nausea" OR "postoperative vomiting" OR "post-operative vomiting" OR PONV OR PDNV OR ((postoperat* OR post-operat* OR postsurg* OR post-surg* OR perioperat* OR anesthe* OR anaesthe*) NEAR/5 (nausea OR vomit* OR emesis OR antiemetic*))):ti,ab,kw #3 #1 AND #2 (results restricted to Trials)	40
Total					146

Selection and Data Collection

Records were deduplicated using digital object identifiers, PubMed identifiers, and normalized titles, followed by manual checking (Appendix 1).

A single reviewer screened titles and abstracts, assessed full texts or full registry records, and extracted data using a piloted form. Study selection, data extraction, risk-of-bias assessment, and Grading of Recommendations Assessment, Development and Evaluation (GRADE) rating were performed by one author (L.B.G.); the second author (A.J.C.F.) contributed to defining the review question and critically revised the manuscript.

Arm-level data were taken from the full published articles for all trials except Seki et al. [4], whose full text could not be obtained; for that trial we used the arm-level counts reported in a previous meta-analysis whose authors had contacted the trial investigators (Grigio et al.) [9], together with the outcome definition in the published trial protocol [13]. For Hyman et al., postoperative counts were taken from the published article and cross-checked against the results posted in ClinicalTrials.gov (NCT02755116); the corrected version of the article was used [3,14]. Trial registries were consulted for every included trial to check prespecified outcomes, including the history of registry changes.

Outcomes

The planned primary outcome was any nausea and/or vomiting within 24 hours after surgery. Because definitions differed, the available primary synthesis pooled each trial's own PONV-related composite within 0-24 hours; in Seki et al., this composite also counted rescue antiemetic administration [4]. Secondary outcomes were nausea, vomiting or retching, rescue antiemetic use (all 0-24 hours), post-discharge outcomes, and sedation. Recovery-room-only outcomes were not pooled with cumulative 0-24-hour outcomes.

Risk of Bias and Certainty

Risk of bias was assessed for each outcome with the Cochrane Risk of Bias (RoB) 2 tool (effect of assignment) [15], answering all signaling questions and applying the published algorithm; answers to all signaling questions are provided in Appendix 4. Certainty of evidence was rated with GRADE [16]. Both were performed by a single reviewer.

Synthesis

Risk ratios (RRs) were calculated from arm-level data. The two olanzapine arms of Abdel-Wahab et al. [7] (5 mg and 10 mg) were combined to avoid double-counting the shared placebo group. Log RRs were pooled with inverse-variance random-effects models, estimating between-study variance by restricted maximum likelihood (REML) and constructing 95% CIs with the modified Hartung-Knapp method (variance scale constrained to at least 1), which is conservative when few trials are available [17,18]. Heterogeneity was described with I² and τ².

Sensitivity analyses were defined during the review, without a protocol, and addressed influential trials (leave-one-out) and discrepant reports in Chen et al. [6] and Abdel-Wahab et al. [7]; the analysis excluding Seki was likewise decided post hoc because of its broader composite definition.

Absolute effects were derived by applying the pooled RR to the observed control-group risk. Funnel plots and small-study tests were not used because no analysis included more than four trials. Analyses were run in Python 3.13 (Python Software Foundation, Fredericksburg, VA) (NumPy, SciPy). Extracted data, screening decisions, search exports, and analysis code are available from the corresponding author upon reasonable request.

Results

Study Selection

The searches identified 146 records (PubMed 25, Embase 81, CENTRAL 40); after removal of 38 duplicates, 108 records were screened, and 31 reports were assessed in full (Figure 1). Eighteen reports were excluded (Appendix 2): five compared olanzapine with standard prophylaxis or another active drug (including the trials of Deb et al. [11] and Ibrahim et al. [19]), two had no concurrent control group, one primarily investigated anxiolysis, one had an unclear comparison, and nine were registered trials that were ongoing, terminated, or completed without available results. One additional report (Seki et al.) was identified through previous systematic reviews. Six trials (14 reports) were included [3-8].

**Figure 1:**
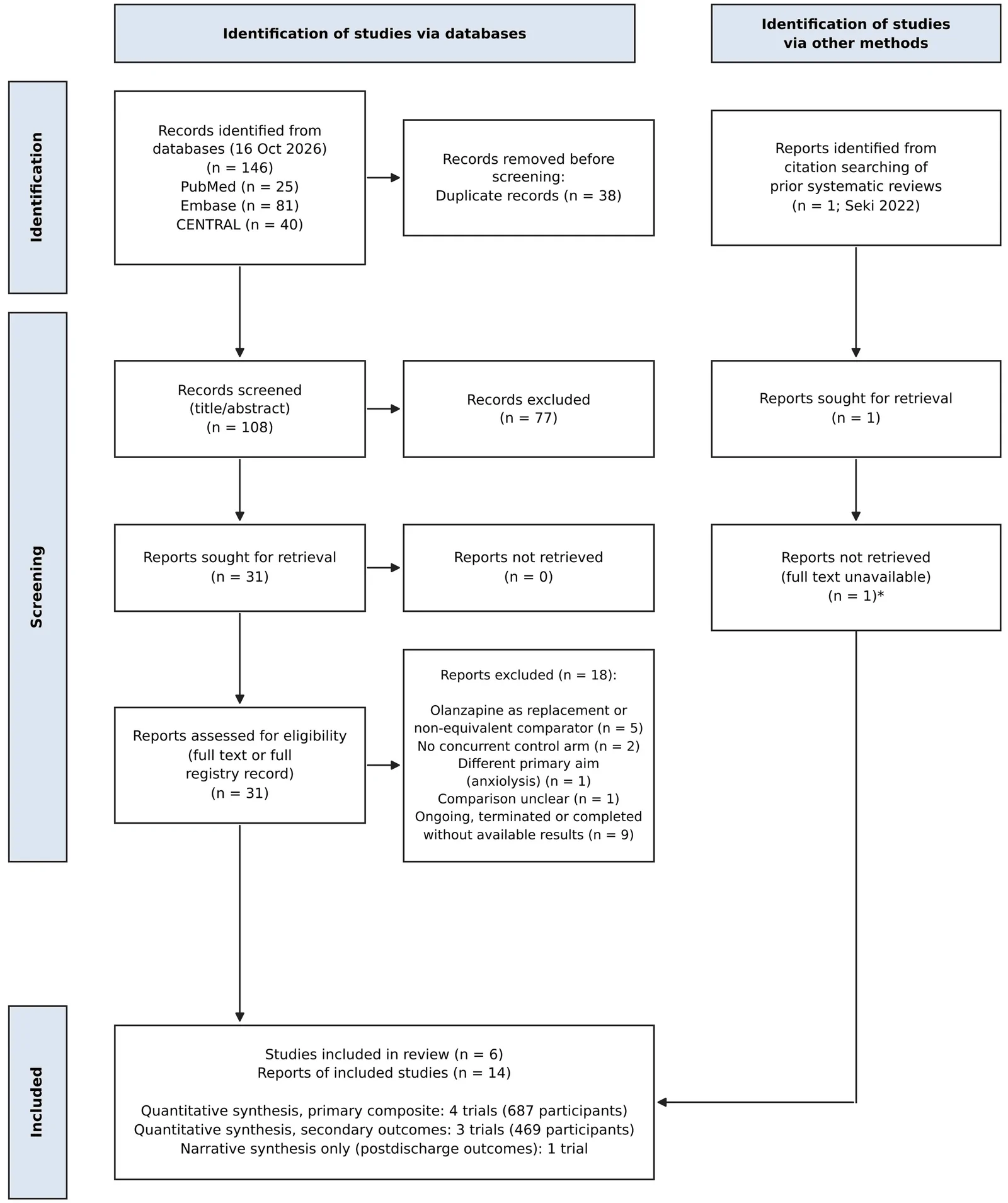
PRISMA 2020 flow diagram of study selection * The full text of Seki et al. [4] could not be retrieved; the trial was included using arm-level data reported in a previous meta-analysis [9] and its published protocol [13]. Its registry record (UMIN000022634) was among the records retrieved from CENTRAL, so the trial is counted once among the six included studies. PRISMA: Preferred Reporting Items for Systematic Reviews and Meta-Analyses; CENTRAL: Cochrane Central Register of Controlled Trials; UMIN: University Hospital Medical Information Network Clinical Trials Registry

Characteristics of Included Trials

Table 2 summarizes the included trials. Five trials enrolled only women, mostly undergoing gynecologic or ambulatory surgery; one enrolled adults with cancer at very high risk of PONV. Olanzapine doses were 5 mg or 10 mg given one to two hours before surgery. Four trials contributed to the primary composite (343 olanzapine, 344 control participants), three to the secondary outcomes, and one (Guzzi et al. [8]) reported only post-discharge and recovery-room outcomes and was synthesized narratively. Arm-level data are shown in Table 3.

**Table 2:** Characteristics of included trials *The protocol reports that the dose was reduced from 10 mg to 5 mg during the trial after drowsiness in three patients [13]. RCT: randomized controlled trial; RR: risk ratio (95% confidence interval); PONV: postoperative nausea and vomiting; POD1: postoperative day 1 (first 24 hours after discharge); ASA: American Society of Anesthesiologists physical status; y: years; N: number of participants; NR: not reported; TIVA: total intravenous anesthesia

Trial	Registry	Design	Population/surgery	N randomized (analyzed)	Olanzapine	Comparator	Background prophylaxis (both groups)	Main findings (0–24 h after surgery unless stated)	Role in synthesis
Hyman 2020 [3]	NCT02755116	Parallel-group RCT, two arms	Women 18–50 y, ambulatory gynecologic/plastic surgery, ≥2 Apfel risk factors [20]; USA	180 consented/randomized; 144 received drug (140)	10 mg	Placebo + same prophylaxis	Dexamethasone + ondansetron; volatile anesthesia	Composite 22/71 vs 35/69 (RR 0.61, 0.40–0.93); postdischarge 10/71 vs 26/69	Primary composite (postoperative 0–24 h, not prespecified); postdischarge narrative
Seki 2022 [4]	UMIN000022634	Multicenter parallel-group RCT, two arms	Women 20–65 y, laparoscopic benign gynecologic surgery, Apfel ≥2; Japan, 5 centers	NR (210)	5 mg*	Placebo + same prophylaxis	Dexamethasone 6.6 mg; sevoflurane	Composite incl. rescue 56/104 vs 72/106 (RR 0.79, 0.64–0.99)	Primary composite (includes rescue antiemetic use); full text unavailable
Grigio 2023 [5]	NCT03631004	Parallel-group RCT, two arms	Adults 18–60 y with cancer, history of chemotherapy-induced nausea/vomiting, Apfel 3–4, medium/major surgery; Brazil	100 (96)	10 mg	Placebo + same prophylaxis	Dexamethasone 4 mg + ondansetron 4 mg; propofol TIVA, mostly epidural analgesia	PONV 12/47 vs 31/49 (RR 0.40, 0.24–0.69)	Primary composite; secondary outcomes
Chen 2024 [6]	ChiCTR2200059115	Parallel-group RCT, two arms	Women 18–75 y, ASA I–II, gynecologic laparoscopy, ≥2 risk factors; China	250 (241)	5 mg	Placebo + same prophylaxis	Dexamethasone 5 mg + granisetron 3 mg; sevoflurane	PONV 10/121 vs 23/120 (RR 0.43, 0.21–0.87)	Primary composite; secondary outcomes
Abdel-Wahab 2025/26 [7]	NCT04718727	Parallel-group RCT, three arms	Women 18–50 y, ASA I–II, elective gynecologic laparoscopy, Apfel >2; Egypt	135, 45 per arm (132)	5 mg and 10 mg (two arms)	Placebo + same prophylaxis	Dexamethasone 8 mg; sevoflurane	Nausea 9/87 vs 23/45 (RR 0.20, 0.10–0.40); vomiting 3/87 vs 11/45	Secondary outcomes (arms combined); composite not reported
Guzzi 2026 [8]	NCT05676294	Parallel-group RCT, two arms	Women 18–50 y, ambulatory surgery	384	5 mg	Placebo + usual prophylaxis	Usual prophylaxis (not restricted)	Nausea POD1 52/191 vs 90/193 (RR 0.58, 0.44–0.77)	Post-discharge outcomes, narrative only

**Table 3:** Arm-level data used in the meta-analyses (0–24 hours after surgery) In Grigio 2023 and Chen 2024, the composite and nausea counts are identical in the source publications. PONV: postoperative nausea and vomiting; N: number of participants analyzed

Outcome	Trial	Olanzapine, events/N	Control, events/N
PONV-related composite	Hyman 2020 [3]	22/71	35/69
PONV-related composite	Seki 2022 [4]	56/104	72/106
PONV-related composite	Grigio 2023 [5]	12/47	31/49
PONV-related composite	Chen 2024 [6]	10/121	23/120
Nausea	Grigio 2023 [5]	12/47	31/49
Nausea	Chen 2024 [6]	10/121	23/120
Nausea	Abdel-Wahab 2025/26 [7]	9/87 (6/43 + 3/44)	23/45
Vomiting or retching	Grigio 2023 [5]	4/47	26/49
Vomiting or retching	Chen 2024 [6]	6/121	9/120
Vomiting or retching	Abdel-Wahab 2025/26 [7]	3/87 (3/43 + 0/44)	11/45
Rescue antiemetic	Grigio 2023 (droperidol) [5]	3/47	18/49
Rescue antiemetic	Chen 2024 (metoclopramide)	4/121	14/120
Rescue antiemetic	Abdel-Wahab 2025/26 (ondansetron) [7]	4/87 (3/43 + 1/44)	8/45

Primary Composite

Across four trials (687 participants), the pooled RR for the study-defined PONV-related composite within 24 hours was 0.59 (95% CI 0.34-1.01; I² = 60%; τ² = 0.066) (Figure 2). With a control-group risk of 46.8%, this corresponds to 194 fewer events per 1,000 patients (from 309 fewer to 5 more). Conventional Wald and standard Hartung-Knapp intervals were narrower (0.42-0.82 and 0.35-0.98, respectively). 

**Figure 2:**
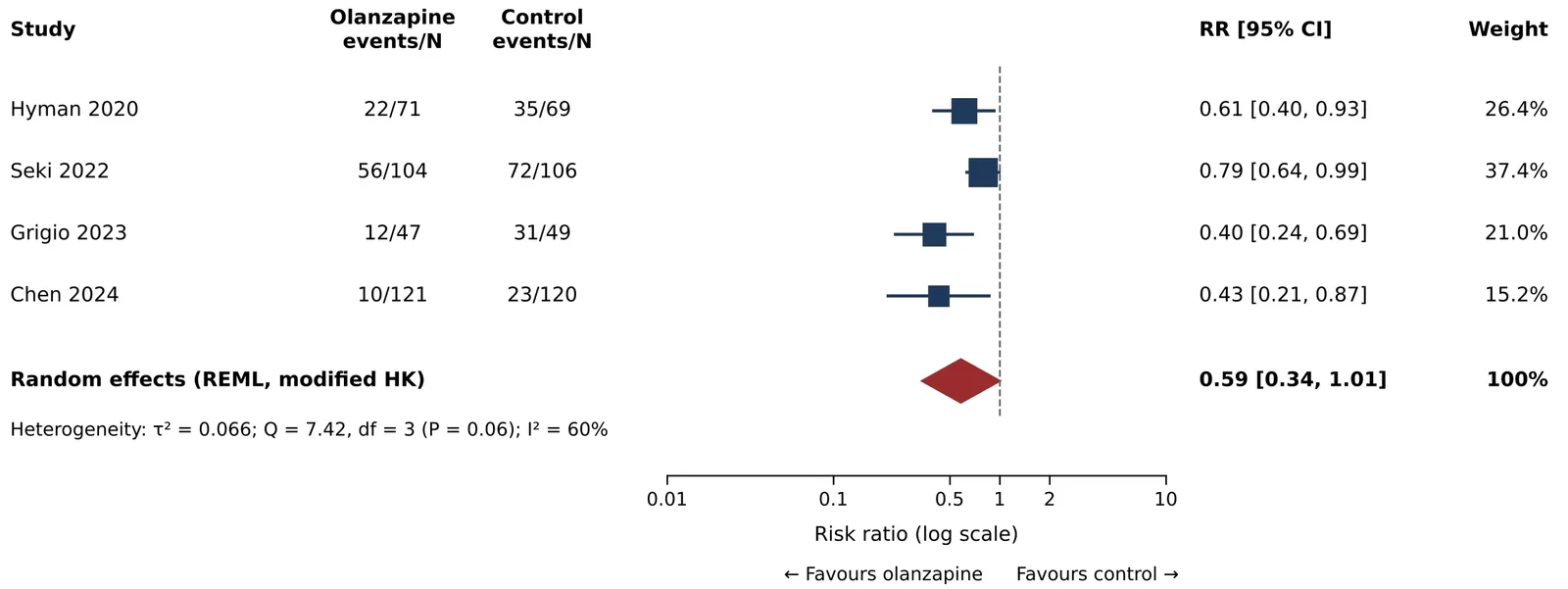
Forest plot of the study-defined PONV-related composite within 24 hours after surgery Four trials [3-6]; I² = 60%, τ² = 0.066. Squares indicate study-specific RRs (size proportional to weight) and horizontal lines indicate their 95% CIs; the diamond indicates the pooled RR from a REML random-effects model with a modified HK 95% CI. The composite of Seki et al. also counted rescue antiemetic use. PONV: postoperative nausea and vomiting; RR: risk ratio; CI: confidence interval; REML: restricted maximum likelihood; HK: Hartung-Knapp; I²: percentage of variability due to heterogeneity; τ²: between-study variance

Two features of this synthesis require emphasis. First, for Hyman et al., the postoperative 0-24-hour outcome is described by the authors as a secondary outcome that was not prespecified; the registry history shows that it was added in July 2020, after publication, when results were posted, whereas the prespecified primary outcome concerned the 24 hours after discharge (10/71 versus 26/69) [3]. Second, Seki et al. counted rescue antiemetic use as part of their prespecified primary composite [4,13].

Sensitivity Analyses

In a post hoc sensitivity analysis excluding Seki (three trials, 477 participants), the RR was 0.50 (0.26-0.98; I² = 0%) (Figure 3). Leave-one-out analyses gave RRs of 0.56 (0.19-1.60) without Hyman, 0.67 (0.35-1.29) without Grigio et al. [5], and 0.62 (0.27-1.40) without Chen. The result therefore depends on which trials are included and should not be interpreted as robust in either direction.

**Figure 3:**
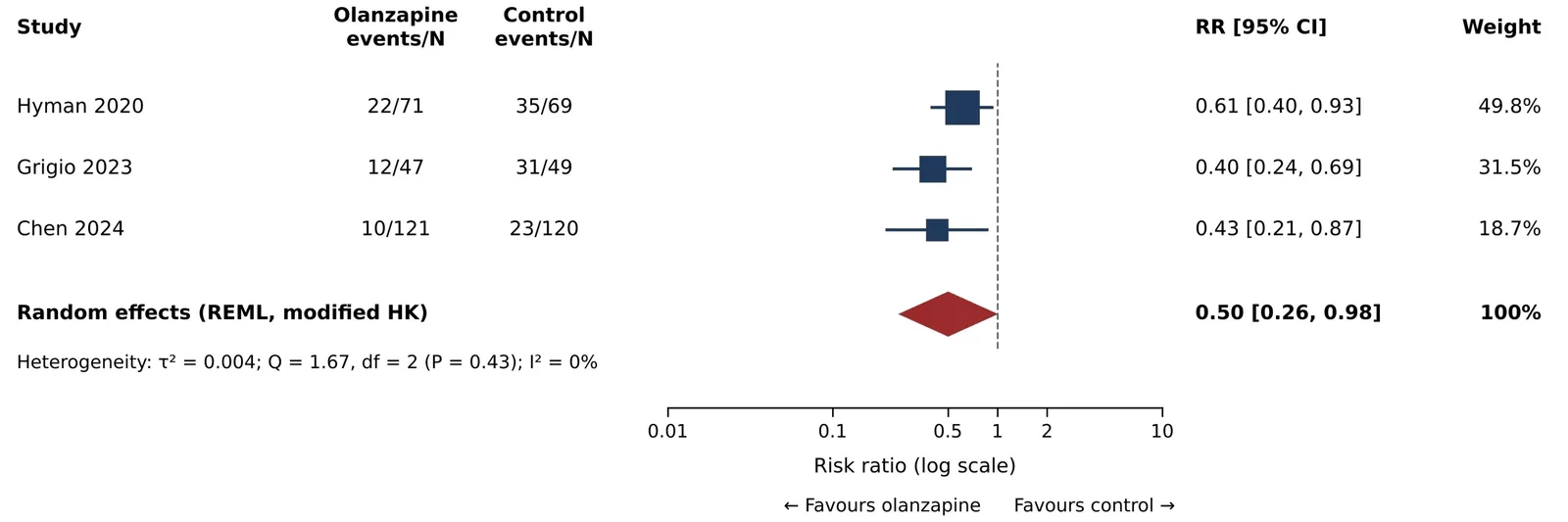
Forest plot of the PONV-related composite excluding Seki et al. (post hoc sensitivity analysis) Three trials [3,5,6]; I² = 0%, τ² = 0.004. Symbols and methods are as in Figure 2. This analysis does not imply that the Seki trial was ineligible. PONV: postoperative nausea and vomiting; RR: risk ratio; CI: confidence interval; REML: restricted maximum likelihood; HK: Hartung-Knapp; I²: percentage of variability due to heterogeneity; τ²: between-study variance

Secondary Outcomes

In three trials (469 participants), olanzapine reduced nausea (RR 0.33, 95% CI 0.12-0.91; I² = 35%) (Figure 4). The effect on vomiting or retching was very imprecise (RR 0.25, 0.03-2.20; I² = 62%) (Figure 5). Olanzapine also reduced rescue antiemetic use (RR 0.24, 0.06-0.98; I² = 0%) (Figure 6). In Chen et al., the nausea and vomiting labels in the supplementary dataset did not match the published table; using the alternative classification gave RRs of 0.36 (0.09-1.42) for nausea and 0.24 (0.05-1.27) for vomiting. In Abdel-Wahab et al., the abstract reported 13 nausea events in the 5-mg arm, whereas the Results text and Table 2 consistently reported 6/43; the main analysis used 9/87 combined events, and using the abstract figure (16/87) gave RR 0.39 (0.19-0.81). Excluding Chen left two trials and produced extremely wide intervals, so the nausea result is not robust to the inclusion of that trial.

**Figure 4:**
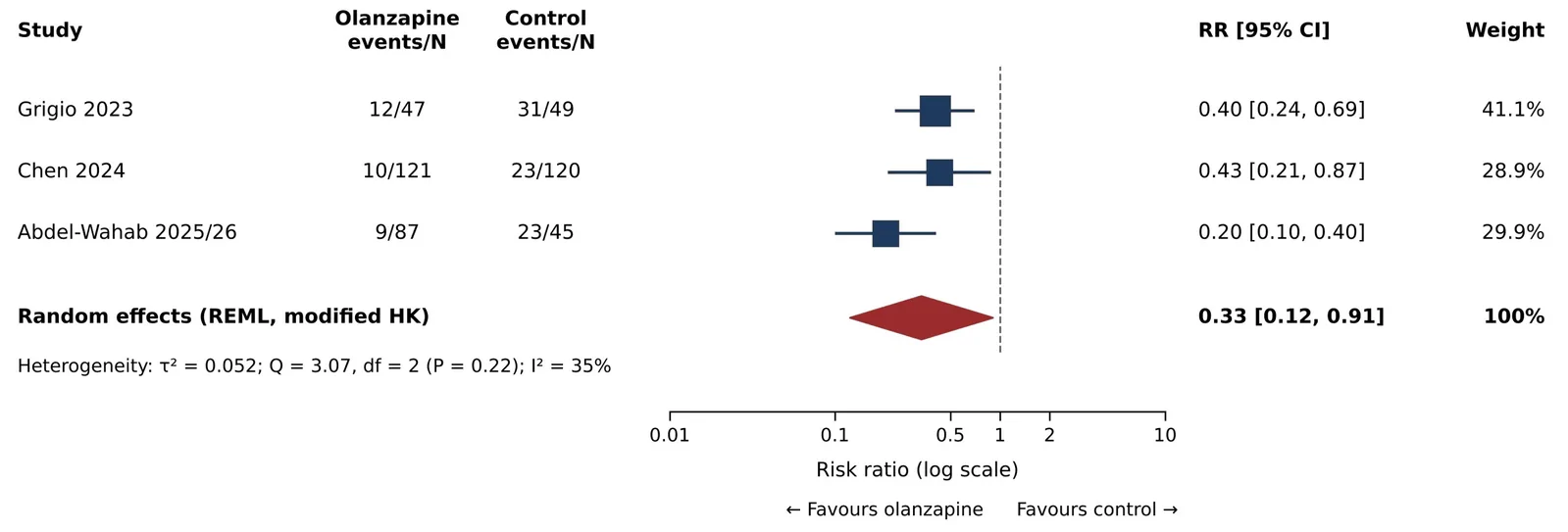
Forest plot of nausea within 24 hours after surgery Three trials [5-7]; I² = 35%, τ² = 0.052. The 5-mg and 10-mg olanzapine arms of Abdel-Wahab et al. were combined against the shared placebo group. Symbols and methods are as in Figure 2. RR: risk ratio; CI: confidence interval; REML: restricted maximum likelihood; HK: Hartung–Knapp; I²: percentage of variability due to heterogeneity; τ²: between-study variance

**Figure 5:**
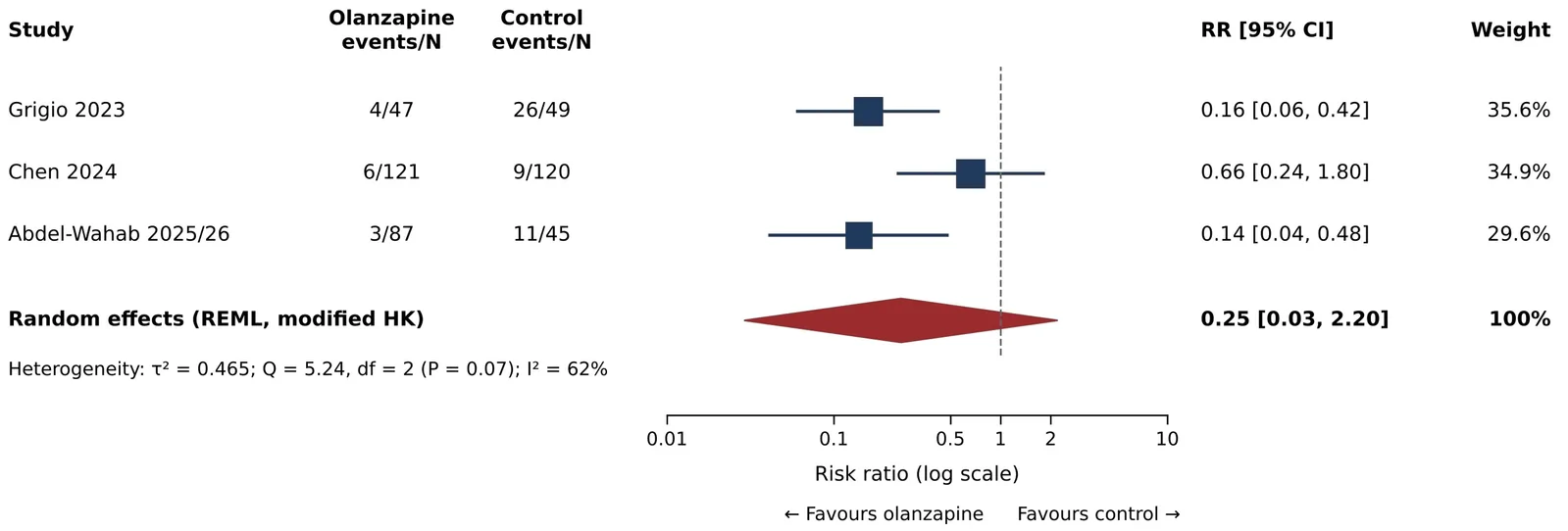
Forest plot of vomiting or retching within 24 hours after surgery Three trials [5-7]; I² = 62%, τ² = 0.465. Olanzapine arms of Abdel-Wahab et al. combined. Symbols and methods as in Figure 2. RR: risk ratio; CI: confidence interval; REML: restricted maximum likelihood; HK: Hartung–Knapp; I²: percentage of variability due to heterogeneity; τ²: between-study variance

**Figure 6:**
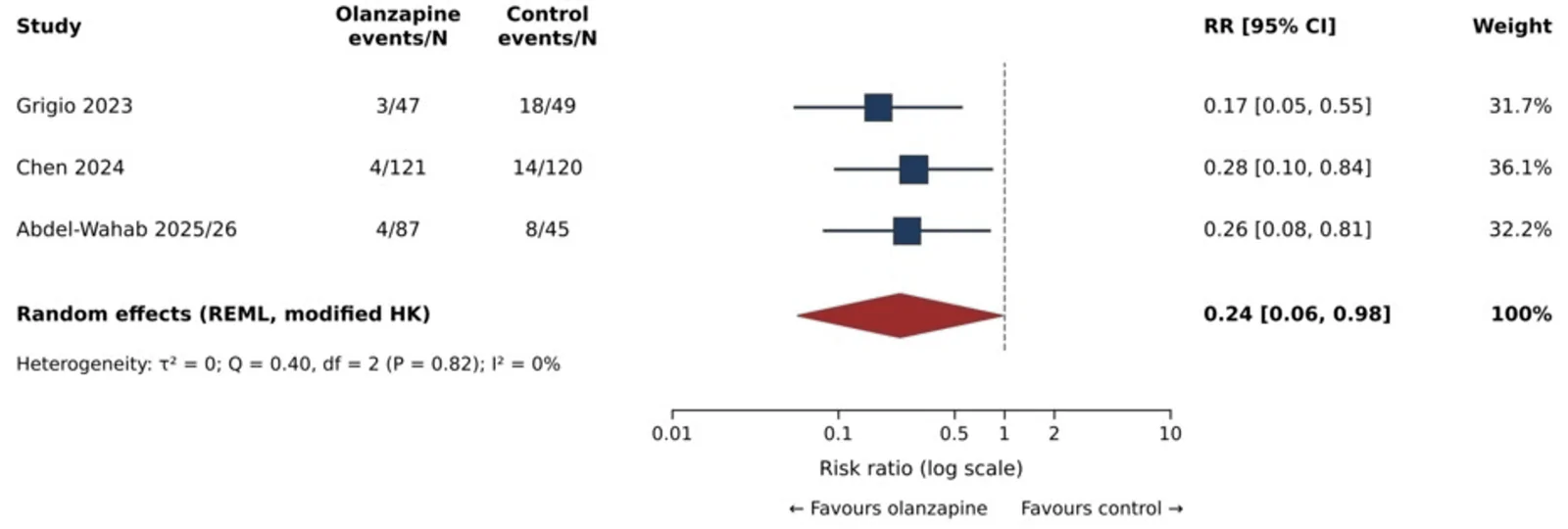
Forest plot of rescue antiemetic use within 24 hours after surgery Three trials [5-7]; I² = 0%, τ² = 0.000. Rescue drugs differed between trials (droperidol, metoclopramide, ondansetron). Olanzapine arms of Abdel-Wahab et al. combined. Symbols and methods as in Figure 2. RR: risk ratio; CI: confidence interval; REML: restricted maximum likelihood; HK: Hartung–Knapp; I²: percentage of variability due to heterogeneity; τ²: between-study variance

Postdischarge Outcomes and Sedation

Guzzi et al. randomized 384 women undergoing ambulatory surgery to olanzapine 5 mg or placebo in addition to routine prophylaxis [8]. Nausea on postoperative day 1 (first 24 hours after discharge) occurred in 52/191 (27%) versus 90/193 (47%) participants (unadjusted RR 0.58, 0.44-0.77), and severe nausea in 15 (7.9%) versus 48 (25%). Hyman et al. reported a reduction in nausea and/or vomiting in the 24 hours after discharge (10/71 versus 26/69) [3]. These postdischarge results were not pooled with postoperative outcomes. Sedation was measured with different instruments and could not be pooled; Hyman et al. reported greater undesired postdischarge sedation with olanzapine 10 mg (median 6 versus 4), and Abdel-Wahab et al. reported higher early sedation scores and more dizziness and headache with 10 mg than with 5 mg or placebo.

Risk of Bias

All 13 outcome-specific assessments were judged to have some concerns (Table 4). The common reason was the exclusion of a small number of randomized participants after allocation (Domain 2) in every trial. Additional concerns related to selection of the reported result (Domain 5) in Hyman et al. (post hoc outcome), Grigio et al. (primary outcome changed in the registry during recruitment and changed back after data collection, although the published outcome matches the original registration) and Abdel-Wahab et al. (registered primary outcome was the composite of nausea and/or vomiting, which was not reported). The assessment of Seki et al. relied on the protocol because the full text was unavailable.

**Table 4:** Risk of bias (RoB 2, effect of assignment), by outcome RoB 2: Cochrane Risk of Bias 2 tool; D1: randomization; D2: deviations from intended interventions; D3: missing outcome data; D4: measurement of the outcome; D5: selection of the reported result. Answers to all signaling questions are provided in Appendix 4.

Trial	Outcome(s)	D1	D2	D3	D4	D5	Overall	Main reasons
Hyman 2020 [3]	Composite	Low	Some concerns	Low	Low	Some concerns	Some concerns	Outcome not prespecified (added to registry after publication); 4 post-allocation exclusions
Seki 2022 [4]	Composite	Low	Some concerns	Low	Low	Low	Some concerns	Modified intention-to-treat analysis; assessed from protocol (full text unavailable)
Grigio 2023 [5]	Composite, nausea, vomiting	Low	Some concerns	Low	Low	Some concerns	Some concerns	4/100 excluded after allocation; registry outcome changed during and after the trial
Grigio 2023 [5]	Rescue antiemetic	Low	Some concerns	Low	Low	Some concerns	Some concerns	As above; rescue use never registered
Chen 2024 [6]	Composite, nausea, vomiting, rescue	Low	Some concerns	Low	Low	Low	Some concerns	9/250 excluded after allocation (conversion to open surgery)
Abdel-Wahab 2025/26 [7]	Nausea, vomiting, rescue	Low	Some concerns	Low	Low	Some concerns	Some concerns	1 post-allocation exclusion; registered primary (nausea and/or vomiting) changed and not reported

Certainty of Evidence

Certainty was very low for the primary composite and for vomiting, and low for nausea and rescue antiemetic use (Table 5). 

**Table 5:** Summary of findings and certainty of evidence (GRADE) Control risk is the pooled observed risk in control groups. Pooled estimates: REML random effects with modified Hartung-Knapp 95% CIs. GRADE: Grading of Recommendations Assessment, Development and Evaluation; RR: risk ratio; CI: confidence interval; PONV: postoperative nausea and vomiting; I²: percentage of variability due to between-study heterogeneity; REML: restricted maximum likelihood

Outcome (0–24 h)	Trials (participants)	RR (95% CI)	Control risk	Absolute effect per 1,000	Certainty	Reasons for downgrading
PONV-related composite	4 (687)	0.59 (0.34–1.01)	468/1,000	194 fewer (309 fewer to 5 more)	Very low	Risk of bias (post hoc outcome in one trial; post-allocation exclusions); inconsistency (I² 60%, differing definitions); imprecision (CI includes no effect)
Nausea	3 (469)	0.33 (0.12–0.91)	360/1,000	239 fewer (316 to 32 fewer)	Low	Risk of bias; imprecision (wide CI, not robust to excluding one trial, discrepant source data)
Vomiting or retching	3 (469)	0.25 (0.03–2.20)	215/1,000	161 fewer (209 fewer to 259 more)	Very low	Risk of bias; inconsistency (I² 62%); very serious imprecision
Rescue antiemetic use	3 (469)	0.24 (0.06–0.98)	187/1,000	143 fewer (176 to 3 fewer)	Low	Risk of bias; imprecision (51 events, upper CI close to no effect)

Discussion

In this systematic review restricted to trials in which olanzapine was the only difference between groups, adjunctive oral olanzapine may reduce postoperative nausea and the need for rescue antiemetics during the first 24 hours, but the evidence for the overall PONV-related composite is very uncertain, and its conservative confidence interval does not exclude no effect. The point estimates consistently favored olanzapine, which agrees with the direction reported in earlier meta-analyses [9,10].

Our estimates differ from those of a previous three-trial meta-analysis (RR 0.62, 95% CI 0.42-0.90) [9] mainly because of trial selection and statistical method. We excluded replacement designs, added two more recent add-on trials, and used modified Hartung-Knapp intervals, which are wider than conventional intervals when only a few trials with heterogeneous results are available [17,18]. We also found that the postoperative outcome of Hyman et al., used in both the previous review and ours, was not prespecified by the trial investigators [3]. Excluding that trial widened the interval considerably, which supports caution.

Heterogeneity in outcome definitions is a central limitation of this evidence base. The trials measured nausea and vomiting at different time points, defined the composite differently, and used different rescue antiemetics and thresholds. The post hoc analysis excluding Seki et al. produced a smaller RR with a confidence interval that narrowly excluded no effect, but analyses selected after seeing the data should not be used to support strong conclusions.

Sedation is the main safety concern. The 10-mg dose was associated with more postdischarge or early postoperative sedation and dizziness in two trials, whereas evidence for dose superiority in efficacy is insufficient. These adverse effects may meaningfully impair patient experience, particularly after ambulatory surgery, and the antiemetic benefit should be weighed against them on an individual basis. Because no trial directly compared the patient-perceived burden of sedation and PONV, future studies should include patient-reported measures of both. A 5-mg dose may therefore be a reasonable choice for future trials. Nine registered trials, several of them completed, had no available results, so the evidence base is likely to change, and reporting bias cannot be excluded.

Limitations

This review has important limitations. Study selection, data extraction, risk-of-bias assessment and GRADE rating were performed by a single reviewer because of limited resources, as no second trained reviewer was available; this increases the risk of errors and subjective judgments, and the findings should therefore be interpreted with caution. The review was not prospectively registered, and eligibility criteria were finalized after the authors were aware of some trial results. The full text of one trial was unavailable, and its data were taken from a previous meta-analysis. Few trials contributed to each analysis, which limits the precision of estimates and precludes assessment of small-study effects. Most participants were women undergoing gynecologic or ambulatory surgery, which limits applicability to other populations; olanzapine may also be useful in major surgery, but this requires confirmation in dedicated trials.

## Conclusions

Adding oral olanzapine to standard antiemetic prophylaxis may reduce postoperative nausea and rescue antiemetic use in adults undergoing general anesthesia, but its effect on the overall incidence of PONV is very uncertain. Adequately powered, prospectively registered trials are needed to validate these findings, with uniform 0-24-hour outcome definitions, separate reporting of nausea, vomiting, and rescue medication, and systematic assessment of sedation, particularly at the 5-mg dose.

## Appendices

Appendix 1

Other Sources

Reference lists of previous systematic reviews [9,10]; trial registry records indexed in CENTRAL (ClinicalTrials.gov and WHO ICTRP sources); registry histories of included trials (ClinicalTrials.gov, ChiCTR), consulted on 16 October 2026.

Deduplication

Records were matched on digital object identifier, PubMed identifier, and normalized title (with first-author check), and matches were reviewed manually. Of 146 records, 38 duplicates were removed, leaving 108 unique records. Record-level screening decisions are available from the corresponding author.

Appendix 2

Reports Assessed in Full and Excluded, With Reasons

**Table 6:** Reports assessed in full and excluded, with reasons CENTRAL: Cochrane Central Register of Controlled Trials; CTRI: Clinical Trials Registry – India; UMIN: University Hospital Medical Information Network Clinical Trials Registry (Japan); ChiCTR: Chinese Clinical Trial Registry; TCTR: Thai Clinical Trials Registry; NCT: ClinicalTrials.gov identifier; vs., versus

Report / registry	Design and comparison	Reason for exclusion
Deb 2024 (Indian J Anaesth) [11]	Olanzapine vs dexamethasone + ondansetron	Olanzapine replaced standard prophylaxis
Painkra 2025 (Int J Life Sci Biotechnol Pharma Res) [21]	Olanzapine vs standard antiemetics	Olanzapine replaced standard prophylaxis
Ibrahim 2013 (Egypt J Anaesth) [19]	Olanzapine vs ondansetron	Olanzapine replaced standard prophylaxis
CTRI/2025/08/093415	Olanzapine vs amisulpride	Non-equivalent active comparator
CTRI/2022/05/042766	Olanzapine vs pregabalin	Non-equivalent active comparator
UMIN000016710	Dose/timing pilot, no control group	No concurrent control arm
UMIN000013908	Safety pilot, no control group	No concurrent control arm
Jiang 2026 (J Bone Joint Surg) [22]	Low-dose olanzapine for perioperative anxiety, arthroplasty	Different primary aim (anxiolysis)
CTRI/2026/07/114695	Three antiemetic combinations	Comparison unclear
TCTR20221027005	Olanzapine vs placebo, gynecologic laparoscopy	Ongoing / no results available
ChiCTR2300076027	Olanzapine vs placebo, breast surgery	Ongoing / no results available
CTRI/2026/05/111427	Olanzapine 5/10 mg + ondansetron vs placebo + ondansetron	Ongoing / no results available
CTRI/2026/08/116166	Olanzapine vs placebo, minimally invasive surgery	Ongoing / no results available
CTRI/2024/03/063683	Preoperative olanzapine vs control	Completed, results not available
CTRI/2025/10/095654	Olanzapine + ondansetron vs ondansetron	Ongoing / no results available
NCT05635227 (GLORIOUS-II)	Factorial trial, cardiac surgery	Ongoing / no results available
UMIN000057772	Olanzapine vs placebo, arthroplasty	Ongoing / no results available
UMIN000030574	Olanzapine vs placebo, gynecologic surgery	Terminated, no results

Appendix 3

PRISMA 2020 Checklist 

**Table 7:** PRISMA 2020 checklist PRISMA: Preferred Reporting Items for Systematic Reviews and Meta-Analyses; RoB 2: Cochrane Risk of Bias 2 tool; GRADE: Grading of Recommendations Assessment, Development and Evaluation; REML: restricted maximum likelihood

Item	Checklist item	Location
1	Title: identify as a systematic review	Title
2	Abstract (PRISMA 2020 for Abstracts)	Abstract
3	Rationale	Introduction & Background
4	Objectives	Introduction & Background, last paragraph
5	Eligibility criteria	Methods, Eligibility criteria
6	Information sources (with dates)	Methods, Information sources; Table 1
7	Full search strategies	Methods, Information sources; Table 1
8	Selection process (reviewers, independence)	Methods, Selection and data collection (single reviewer)
9	Data collection process	Methods, Selection and data collection
10a	Outcomes sought	Methods, Outcomes
10b	Other variables	Methods; Table 2
11	Risk of bias assessment	Methods, Risk of bias and certainty; Appendix 4
12	Effect measures	Methods, Synthesis (risk ratio)
13a	Synthesis eligibility	Methods, Synthesis; Table 3
13b	Data preparation (combining arms)	Methods, Synthesis
13c	Tabulation and display	Tables 2–3; Figures 2–6
13d	Synthesis methods	Methods, Synthesis (REML, modified Hartung–Knapp)
13e	Heterogeneity exploration	Methods, Synthesis (I², τ²; leave-one-out)
13f	Sensitivity analyses	Methods, Synthesis; Results
14	Reporting bias assessment	Methods, Synthesis (not feasible; registries checked)
15	Certainty assessment	Methods, Risk of bias and certainty (GRADE)
16a	Results of search and selection	Results, Study selection; Figure 1
16b	Excluded studies with reasons	Results, Study selection; Appendix 2
17	Study characteristics	Table 2
18	Risk of bias in studies	Table 4; Appendix 4
19	Results of individual studies	Table 3; Figures 2–6
20a	Characteristics and risk of bias of syntheses	Results; Tables 4–5
20b	Results of syntheses	Results; Figures 2–6
20c	Heterogeneity investigations	Results, Primary composite (leave-one-out)
20d	Sensitivity analyses	Results
21	Reporting biases	Results, Risk of bias; Discussion (unpublished registered trials)
22	Certainty of evidence	Table 5
23a	Interpretation	Discussion
23b	Limitations of evidence	Discussion
23c	Limitations of review process	Discussion, Limitations
23d	Implications	Discussion; Conclusions
24a	Registration	Methods (not registered)
24b	Protocol	Methods (no protocol)
24c	Amendments	Methods (Outcomes; Synthesis)
25	Support/funding	Additional Information
26	Competing interests	Additional Information
27	Availability of data, code and materials	Methods, Synthesis

Appendix 4

RoB 2 Signaling Question Answers (Effect of Assignment) 

**Table 8:** RoB 2 signaling question answers (effect of assignment) Answers in question order within each domain (Y: yes; PY: probably yes; PN: probably no; N: no; NI: no information; NA: not applicable). D1: 1.1–1.3; D2: 2.1–2.7; D3: 3.1–3.4; D4: 4.1–4.5; D5: 5.1–5.3. All 13 overall judgments were “some concerns”. RoB 2: Cochrane Risk of Bias 2 tool; D1–D5: risk-of-bias domains as defined in Table 4.

Trial	Outcome	D1	D2	D3	D4	D5
Hyman 2020 [3]	PONV composite, 0–24 h after surgery (not prespecified)	Y/Y/PN	N/N/NA/NA/NA/PN/PN	PN/N/N/NA	N/N/PN/NA/NA	N/PN/PN
Seki 2022 [4]	Pragmatic composite (nausea, vomiting or rescue antiemetic)	Y/Y/NI	N/N/NA/NA/NA/PN/PN	NI/NI/PN/NA	N/N/N/NA/NA	Y/N/PN
Grigio 2023 [5]	PONV composite, 0–24 h	Y/Y/PN	N/N/NA/NA/NA/PN/PN	PY/NA/NA/NA	N/N/PN/NA/NA	PN/PN/PN
Grigio 2023 [5]	Nausea, 0–24 h	Y/Y/PN	N/N/NA/NA/NA/PN/PN	PY/NA/NA/NA	N/N/PN/NA/NA	PN/PN/PN
Grigio 2023 [5]	Vomiting/retching, 0–24 h	Y/Y/PN	N/N/NA/NA/NA/PN/PN	PY/NA/NA/NA	N/N/PN/NA/NA	PN/PN/PN
Grigio 2023 [5]	Rescue antiemetic (droperidol)	Y/Y/PN	N/N/NA/NA/NA/PN/PN	PY/NA/NA/NA	N/N/PN/NA/NA	PN/PN/PN
Chen 2024 [6]	Composite nausea and/or vomiting, 24 h	Y/PY/PN	N/N/NA/NA/NA/N/PN	PY/NA/NA/NA	N/N/PN/NA/NA	PY/PN/PN
Chen 2024 [6]	Nausea, 24 h	Y/PY/PN	N/N/NA/NA/NA/N/PN	PY/NA/NA/NA	N/N/PN/NA/NA	PY/PN/PN
Chen 2024 [6]	Vomiting/retching, 24 h	Y/PY/PN	N/N/NA/NA/NA/N/PN	PY/NA/NA/NA	N/N/PN/NA/NA	PY/PN/PN
Chen 2024 [6]	Rescue antiemetic (metoclopramide)	Y/PY/PN	N/N/NA/NA/NA/N/PN	PY/NA/NA/NA	N/N/PN/NA/NA	PY/PN/PN
Abdel-Wahab 2025/26 [7]	Nausea, 24 h	Y/PY/N	N/N/NA/NA/NA/PN/PN	PY/NA/NA/NA	N/N/PN/NA/NA	PN/PN/PN
Abdel-Wahab 2025/26 [7]	Vomiting, 24 h	Y/PY/N	N/N/NA/NA/NA/PN/PN	PY/NA/NA/NA	N/N/PN/NA/NA	PN/PN/PN
Abdel-Wahab 2025/26 [7]	Rescue antiemetic (ondansetron)	Y/PY/N	N/N/NA/NA/NA/PN/PN	PY/NA/NA/NA	N/N/PN/NA/NA	PN/PN/PN
